# High throughput spatial immune mapping reveals an innate immune scar in post-COVID-19 brains

**DOI:** 10.1007/s00401-024-02770-6

**Published:** 2024-07-25

**Authors:** Marius Schwabenland, Dilara Hasavci, Sibylle Frase, Katharina Wolf, Nikolaus Deigendesch, Joerg M. Buescher, Kirsten D. Mertz, Benjamin Ondruschka, Hermann Altmeppen, Jakob Matschke, Markus Glatzel, Stephan Frank, Robert Thimme, Juergen Beck, Jonas A. Hosp, Thomas Blank, Bertram Bengsch, Marco Prinz

**Affiliations:** 1 Institute of Neuropathology, Faculty of Medicine, University of Freiburg, Breisacher Str. 64, 79106 Freiburg, Germany; 2 Department of Neurology and Neuroscience, University Medical Center, Faculty of Medicine, University of Freiburg, Freiburg, Germany; 3 Department of Neurosurgery, University Medical Center Freiburg, Freiburg, Germany; 4 Institute of Medical Genetics and Pathology, University Hospital Basel, University of Basel, Basel, Switzerland; 5 Max Planck Institute for Immunobiology and Epigenetics, 79108 Freiburg, Germany; 6 Institute of Pathology, Cantonal Hospital Baselland, Liestal, Switzerland; 7 University of Basel, Basel, Switzerland; 8 Institute of Legal Medicine, University Medical Center Hamburg-Eppendorf, Hamburg, Germany; 9 Institute of Neuropathology, University Medical Center Hamburg-Eppendorf, Hamburg, Germany; 10 Division of Neuropathology, Institute of Medical Genetics and Pathology, University Hospital Basel, University of Basel, Basel, Switzerland; 11 Clinic for Internal Medicine II, Gastroenterology, Hepatology, Endocrinology, and Infectious Disease, Faculty of Medicine, University Medical Center Freiburg, Freiburg, Germany; 12 Signalling Research Centres BIOSS and CIBSS, University of Freiburg, Freiburg, Germany

**Keywords:** COVID-19, SARS-CoV-2, Microglia, Imaging mass cytometry, Long-COVID, Post-COVID condition, PCC, Post-acute COVID syndrome, PACS, Neuro-long-COVID-19

## Abstract

The underlying pathogenesis of neurological sequelae in post-COVID-19 patients remains unclear. Here, we used multidimensional spatial immune phenotyping and machine learning methods on brains from initial COVID-19 survivors to identify the biological correlate associated with previous SARS-CoV-2 challenge. Compared to healthy controls, individuals with post-COVID-19 revealed a high percentage of TMEM119^+^P2RY12^+^CD68^+^Iba1^+^HLA-DR^+^CD11c^+^SCAMP2^+^ microglia assembled in prototypical cellular nodules. In contrast to acute SARS-CoV-2 cases, the frequency of CD8^+^ parenchymal T cells was reduced, suggesting an immune shift toward innate immune activation that may contribute to neurological alterations in post-COVID-19 patients.

**Supplementary Information:**

The online version contains supplementary material available at 10.1007/s00401-024-02770-6.

## Introduction

Acute COVID-19 is frequently accompanied by neurological symptoms, and a subset of individuals experiences persistent neurological post-COVID-19 manifestations, referred to as Post-COVID condition (PCC), Post-Acute COVID Syndrome (PACS) or Neuro-Long-COVID-19 [4]. However, the underlying pathogenesis for this phenomenon is largely unclear. Recent studies looking into the blood composition of patients with Long-COVID detected altered composition of immune cells [10, 17, 29], increased peripheral complement activation [3], dysregulation of markers related to blood clotting and coagulation [26], or exaggerated humoral response directed against SARS-CoV-2 [10] with unclear relevance for the central nervous system (CNS). Regarding the central nervous system, some studies encompassed large clinical datasets [2, 13, 24, 30] or advanced imaging techniques. For example, repeated magnetic resonance brain imaging revealed significant longitudinal effects in individuals previously infected with SARS-CoV-2, including a greater reduction in gray matter thickness and global brain size [5] that may be a consequence of T-cell-mediated neuroinflammation previously described in acutely affected COVID-19 brains [12, 22, 28]. Studies involving patients or patient samples of subacute, late-stage COVID-19 indicate an altered glucose metabolism [9] and type I interferon response [18]. Despite these proposed mechanisms, the cellular and molecular processes that are affected in the brains of long-term post-COVID-19 patients are largely missing.

## Methods

### Specimen collection

Formaldehyde-fixed paraffin-embedded (FFPE) central nervous system samples were obtained from autopsies at the University Medical Center in Freiburg, the Institute of Pathology, University of Basel, Institute of Legal Medicine at the University Medical Center Hamburg-Eppendorf and the Institute of Neuropathology at the University Medical Center Hamburg-Eppendorf. Post-COVID-19 patients had a COVID-19 unrelated cause of death, which was assessed by thorough assessment of the medical records, interviews with relatives, and meticulous autopsy findings. Patients had reported full recovery from COVID-19 and showed no signs of COVID-related symptoms before their passing. In particular, no long-term neurological deficits after exposure to SARS-CoV-2 have been reported. Patients were typically tested negative for SARS-CoV-2 after their COVID-19 infection during their lifetime. Additional testing for SARS-CoV-2 was performed for six patients during autopsy, and the test results were all negative. Patient characteristics are provided in Supplementary Table 1. Lumbar punctures of Neuro-Long-COVID-19 patients were performed at the Department of Neurology and Neuroscience at the University Medical Center Freiburg (Supplementary Table 2). Cerebrospinal fluid from patients with idiopathic intracranial hypertension served as controls. The analyses were performed with the approval of the institutional review boards (Ethics Committee of the University of Freiburg: 211/20, 10008/09; Ethics Committee of the Hamburg Chamber of Physicians: 2020-10353-BO-ff, PV7311; Ethics Committee of Northwestern and Central Switzerland: 2020-00629). The study was performed in agreement with the principles expressed in the Declaration of Helsinki and its amendments.

### Immunohistochemistry

The immunohistochemical reactions (chromogenic immunohistochemistry) for CD8a, CD4, CD20, SARS-CoV spike glycoprotein, APP, and Alpha-Syn on 3 µm-thick FFPE sections were performed using the EnVision Flex Kit (DAKO, Agilent, cat. # K8000) and a DAKO Autostainer Link 48 system. EnVision low pH tissue pre-treatment was used for CD8a (Dako, cat. # IR623, RTU), APP (Millipore, cat. # MAB348, 1:2000), SARS-CoV spike glycoprotein (abcam, cat. # ab272420, 1:100), and Alpha-Syn (BioSB, cat. # BSB3291, RTU). EnVision high pH tissue pre-treatment was used for CD20 (DAKO, cat. # IR604, RTU) and CD4 (DAKO, cat. # IR649, RTU). EnVision Flex Mouse Linker (DAKO, cat. # K800221-2) was applied for CD4 immunohistochemistry.

Chromogenic Iba1 immunohistochemistry of 3 µm-thick FFPE sections was performed using the labeled streptavidin–biotin (LSAB) method as previously described [20, 21]. Slides were deparaffinized in Xylene and cooked in EnVision low pH antigen retrieval buffer for 40 min. Endogenous tissue peroxidase was quenched in 3% hydrogen peroxidase (Carl Roth, cat. # 8070.1) for 10 min. Samples were then blocked with 10% normal goat serum (SouthernBiotech, cat. # 0060-01), 1% Triton X-100 (Sigma, cat. # T8787-100ML) in TRIS buffer (EnVision Flex Wash Buffer, DAKO, cat. # K8000) for 1 h. The incubation with Iba1 primary antibody (abcam, cat. # 178846, 1:1000 in the blocking solution) was performed at room temperature overnight. After three washes with TRIS buffer, the slides were incubated with goat anti-rabbit secondary antibody (SouthernBiotech, cat. # 4050-08, 1:300 in the blocking solution) for 45 min. Slides were then washed three times. Streptavidin-HRP (SouthernBiotech, cat. # 7105-05) was diluted 1:1000 in TRIS buffer and added for 45 min. Specimens were then rinsed in TRIS buffer three times and incubated with DAB solution (1 drop EnVision Flex DAB Chromogen per 1 ml EnVision Flex Substrate Buffer).

All sections were counterstained with Gill’s Hematoxylin solution (Sigma, cat. 1051750500) and Vitro-Clud (R. Langenbrinck GmbH, cat. # 04-0001) was used as mounting medium. Imaging was performed on a Zeiss Axioscan 7 system equipped with a 20× objective.

### Imaging mass cytometry

Imaging Mass Cytometry was conducted as reported previously [1, 22]. In short, antibodies were conjugated to lanthanide metals using the Maxpar X8 antibody labeling kit. 4 µm thick formaldehyde-fixed paraffin-embedded (FFPE) sections were deparaffinized and cooked in EnVision FLEX Target Retrieval Solution High pH (DAKO, cat. # K8000) for 40 min. Sections were blocked using SuperBlock Blocking Buffer (ThermoFisher, cat. # 37581). The slides were then incubated with the antibody mix (Supplementary Table 3) in 0.5% BSA, 1% Triton-X-100 in TRIS at room temperature overnight. Iridium Cell-ID intercalator (Fluidigm, cat. # 201192A) was used to visualize DNA and applied for 30 min. The measurement was conducted using the Hyperion Imaging Mass Cytometry system (Fluidigm). For the IMC measurements, regions of interest (ROIs) were determined by Iba1 immunohistochemistry on a consecutive section. Areas with most prominent microglia nodules (if present) were selected.

**Fig. 1:**
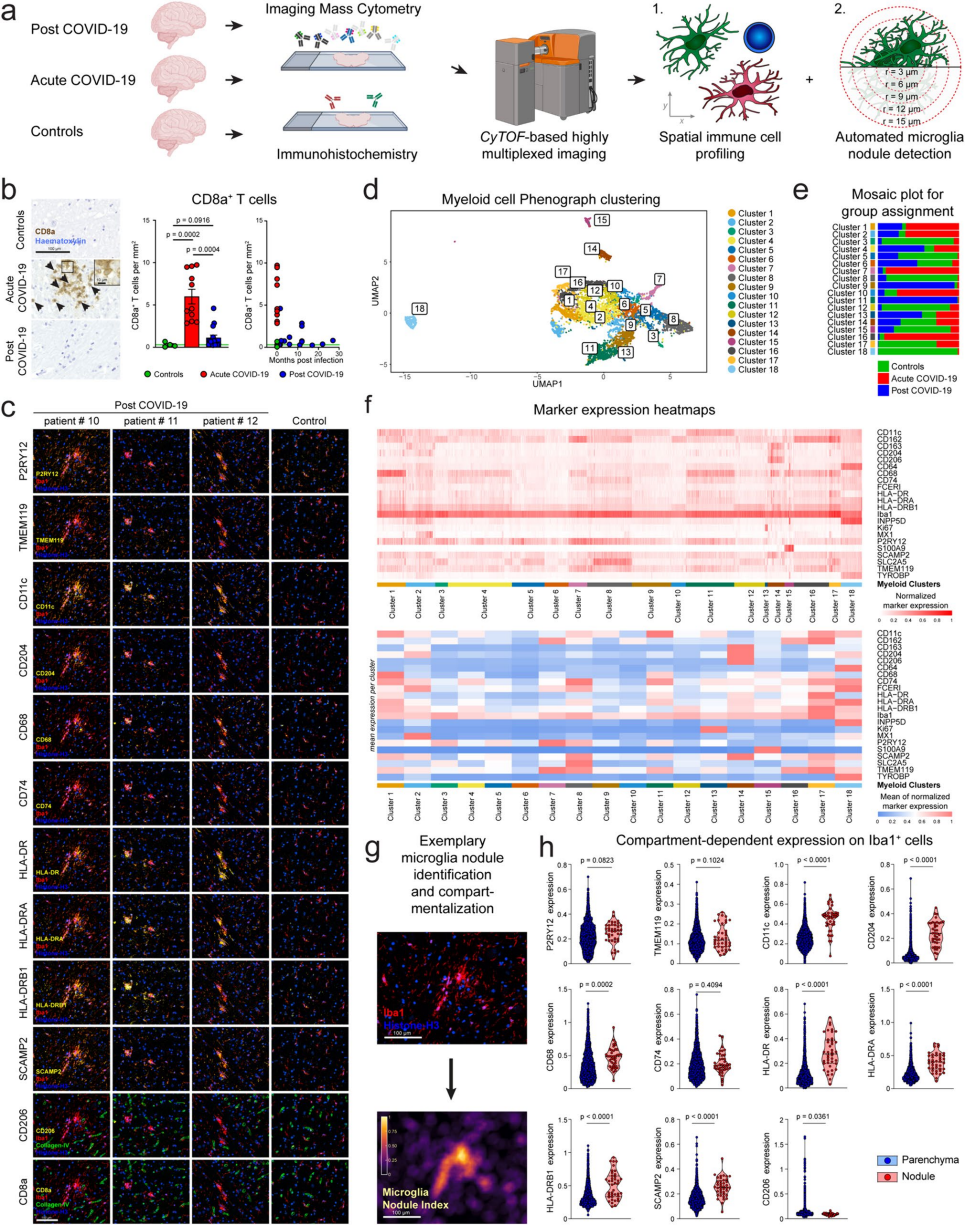
Innate rather than adaptive immune activation in defined compartments of post-COVID-19 brains. **a** Experimental workflow. Autopsy tissue slices from the upper medulla of 15 initial COVID-19 survivors with SARS-CoV-2-unrelated causes of death (post-COVID-19), 11 acute COVID-19 and 4 SARS-CoV-2 naïve control patients were analyzed by comprehensive neuropathological analyses. *CyTOF* cytometry by time of flight. **b** Left: representative immunohistochemistry for CD8a (brown) in the upper medulla oblongata of controls, acute COVID-19 patients (cells are highlighted by arrow heads) and post-COVID-19 patients. Counterstaining with haematoxylin. Scale bar: 100 µm. Middle: quantification thereof. Each symbol represents one patient. Bars represent means ± SEM. *P* values were determined using Brown-Forsythe and Welsh ANOVA test with Dunnett’s T3 multiple comparisons test. Right: T-cell numbers over different time points post-infection. Acute COVID-19 patients and controls are shown at 0 months post-infection. Each symbol represents one patient. Green line indicates the mean of controls. **c** Representative images depicting imaging mass cytometry of the medulla oblongata of three post-COVID-19 patients and one age-matched control. Scale bar: 100 µm. **d** UMAP visualization shows Phenograph clustering of myeloid cells in post-COVID-19 brains. Myeloid cells from both the frontal cortex and the upper medulla oblongata were analyzed. **e** Mosaic plot representing the contribution of identified myeloid cell clusters to each control, acute COVID-19, and post-COVID-19 brain samples. Myeloid cells from the frontal cortex and the upper medulla oblongata were analyzed. **f** Heatmaps visualizing the protein expression profiles of the different myeloid clusters. One column represents the normalized expression of one cell in the upper panel. In the lower panel, the mean expression per cluster is shown. **g** Representative visualization of the microglia nodule index calculated based on Iba1 signal across a 15 µm radius. A threshold of 0.5 was applied for the compartmentalization into parenchymal and nodule-associated myeloid cells. **h** Violin plots depicting the normalized expression of the indicated markers on Iba1^+^ myeloid cells in the parenchymal (blue) and subtissular nodule compartment (red) in the medulla of post-COVID-19 individuals. Student’s t test was applied. *P* values are indicated. Each symbol represents one cell

Image segmentation was performed based on the IMCSegmentationPipeline [27]. Image visualization was performed using MCD Viewer v1.0.560.6. An expression threshold of > = 2 and area > = 20 was used for the gating of Iba1 myeloid cells (Fig. 1D). For PhenoGraph clustering, the expression values of CD11c, CD162, CD163, CD204, CD206, CD64, CD68, FCERI, HLA-DR, HLA-DRA, HLA-DRB1, Iba1, INPP5D, Ki67, MX1, P2RY12, S100A9, SCAMP2, SLC2A5, TMEM119, and TYROBP were used. For Fig. 1F, a min–max scaling was performed for normalization. In Fig. 1H, expression values were normalized to Iba1 signal intensity. Compartments (parenchyma vs. nodule) were based on the microglia nodule index [22] (defined as the coverage of Iba1 signal in a 15 µm radius) and a threshold >  = 0.5.

### Cerebrospinal fluid pre-treatment

Cerebrospinal fluid samples were centrifuged at 2000*g* for 10 min at + 4 °C. The cell-free supernatant was snap-frozen and kept at − 80 °C.

### Enzyme-linked immunosorbent assays (ELISA)

After thawing CSF samples on ice, enzyme-linked immunosorbent assays (YKL-40: Invitrogen, cat. # EHCHI3L1; TREM2: Invitrogen, cat. # EH464RB; CD14: Invitrogen, cat. # EHCD14; NF-light: Tecan, cat. # UD51001) were performed according to the manufacturer’s instructions. A Spark multimode microplate reader (Tecan) was used for measurements.

### Metabolomics

Targeted metabolomics analysis was conducted following established procedures outlined in the previous studies [15] and involved the extraction of samples using a precooled extraction solution (80:20 methanol LC–MS grade: Milli-Q water). Quantification of targeted metabolites through LC–MS was performed on an Agilent 1290 Infinity II UHPLC coupled with an Agilent 6495 QQQ-MS operating in MRM mode. MRM settings were optimized individually for all compounds using pure standards. Isotopically labeled yeast extract (ISOtopic Solutions, Vienna, Austria) was spiked into all samples as internal standard for identification of correct peaks and compensation of matrix effects. LC separation was performed as published previously [19]. Briefly, a Waters Atlantis Premier BEH ZHILIC column (100 × 2.1 mm, 1.7 µm particles) was used, buffer A was 20 mM ammonium carbonate and 5 µM medronic acid in milliQ H_2_O and buffer B was 90:10 acetonitrile:buffer A and the solvent gradient was from 95 to 55% buffer B over 18 min. Flow rate was 150 µL/min, column temperature was 40 °C, autosampler temperature was 5 °C, and injection volume was 2 µL. Data processing was performed using the R package automRm [6]. Spearman correlations were performed as reported previously [22].

### Statistical analyses

Statistical analyses and visualizations were performed using GraphPad Prism 9.5.1. The statistical tests are mentioned in the figure legends. *P* values are stated in the figures.

## Results

### Innate rather than adaptive immune activation in defined compartments of post-COVID-19 brains

In a multicenter study, we collected brains from 15 individuals that experienced a previously confirmed SARS-CoV-2 infection, recovered fully but died due to reasons unrelated to COVID-19 up to 27 months after viral infection (Supplementary Table 1). Brains from four healthy controls and 11 acute COVID-19 cases were included for comparison. First, all samples underwent a comprehensive COVID-19 centered neuropathological analysis by board-certified neuropathologists as previously described [12]. Some samples (patients # 10, 11, and 12) were subsequently analyzed by single-cell-based immune phenotyping by cytometry-by-time-of-flight-(CyTOF)-based imaging mass cytometry (IMC) that allows detailed spatial profiling of single immune cells in the diseased CNS (Fig. 1a) [22]. Surprisingly, and in contrast to CNS specimens from acute cases, post-COVID-19 brain revealed only very few parenchymal CD8a^+^ T cells that were numerically almost compatible to controls (Fig. 1b). Additionally, we have also examined CD4^+^ T cells and CD20^+^ B cells (Supplementary Fig. 1) and found no significant differences in parenchymal cell counts between controls and post-COVID-19 cases. In contrast, using high-dimensional, 40 marker-based IMC analyses of samples from the frontal cortex and the medulla, we could identify microglia cells with positive signal in the channels for the microglial markers P2RY12 and TMEM119, accompanied by signals in the channels for myeloid cell molecules CD11c, CD68, CD204, and SCAMP2, along with the induction of the MHC class II-related proteins HLA-DR, HLA-DRA, and HLA-DRB1 (Fig. 1c). We next employed a supervised machine learning approach to segment the images and to extract single-cell expression data based on the intensity of each marker in the respective channel. After gating for the pan-myeloid marker ionized calcium-binding adaptor molecule 1 (Iba1), cells were clustered using the PhenoGraph algorithm (Fig. 1d). This approach identified a total of 18 clusters, as shown in a uniform manifold approximation and projection (UMAP) representing diverse myeloid cells, such as microglia (e.g., P2RY12^+^, TMEM119^+^ clusters 1, 2, 7, 8, and 11), perivascular macrophages (CD163^+^, CD204^+^, and CD206^+^ cluster 14), and monocytes (S100A9^+^ cluster 15). As depicted in mosaic plots, we identified clusters enriched for specific stages following SARS-CoV-2 infection, such as cluster 11 that was enhanced in post-COVID-19, and cluster 7 that was prominent in acute COVID-19 patients (Fig. 1e). Marker expression heat maps depict the varying marker expression profiles leading to distinct clusters (Fig. 1f). For example, microglia cells in cluster 1 exhibited strong positivity for the lysosomal activation marker CD68 and were mostly observed in samples from acute and post-COVID-19 patients, with minimal representation in control samples. Cluster 11, characterized by elevated integrin alpha x (CD11c) expression indicative of an activated microglial cell state, was mostly found in post-COVID-19 patients. Cluster 9 was also found in post-COVID-19 patients and was characterized by a moderate CD11c expression. Cluster 16 cells were primarily seen in acute cases, expressed the lysosomal activation marker CD68, and the MHC class-II-related molecules CD74, HLA-DRA, and HLA-DRB1. Myeloid cluster 14 that was shared across all patient groups expressed the markers CD206 and scavenger receptor cysteine-rich type 1 protein M130 (CD163) indicative of perivascular macrophages. Notably, Iba1^+^P2RY12^+^TMEM119^+^ microglia were found to be assembled in characteristic clusters, known as microglia nodules, that we defined as ≥ 50% coverage of the Iba1 signal in a 15 µm radius. Microglia nodules are usually considered morphological hallmarks of chronic neuropathological processes, such as viral encephalopathies, axonal damages, or neurodegenerative changes [22].

We next explored the spatial distribution of marker expression within single brain sections. To achieve this, we compartmentalized the dataset into regions containing microglia nodules and non-microglia-nodule areas, utilizing our previously developed microglia nodule index (Fig. 1g) [22]. We then compared the normalized marker expression of cells within nodules to those outside nodules in the medulla of post-COVID-19 patients (Fig. 1h). No statistically significant differences were observed for expression of P2RY12 or TMEM119 on microglia localized inside or outside the nodules allowing to unequivocally determine their cellular identity. Importantly, spatial analysis revealed a higher expression of CD11c, CD68, CD204, HLA-DR, HLA-DRA, HLA-DRB1, and SCAMP2 on P2RY12^+^TMEM119^+^ microglia within nodules. No differences were observed for CD74 and CD206. Collectively, these data identify the innate rather than the adaptive immune system as main functional player in post-COVID-19 brains and highlight the microglia nodule compartment as the key site of local tissue immune responses.

### Persistent activation of microglia characterizes the CNS of post-COVID-19 patients

**Fig. 2:**
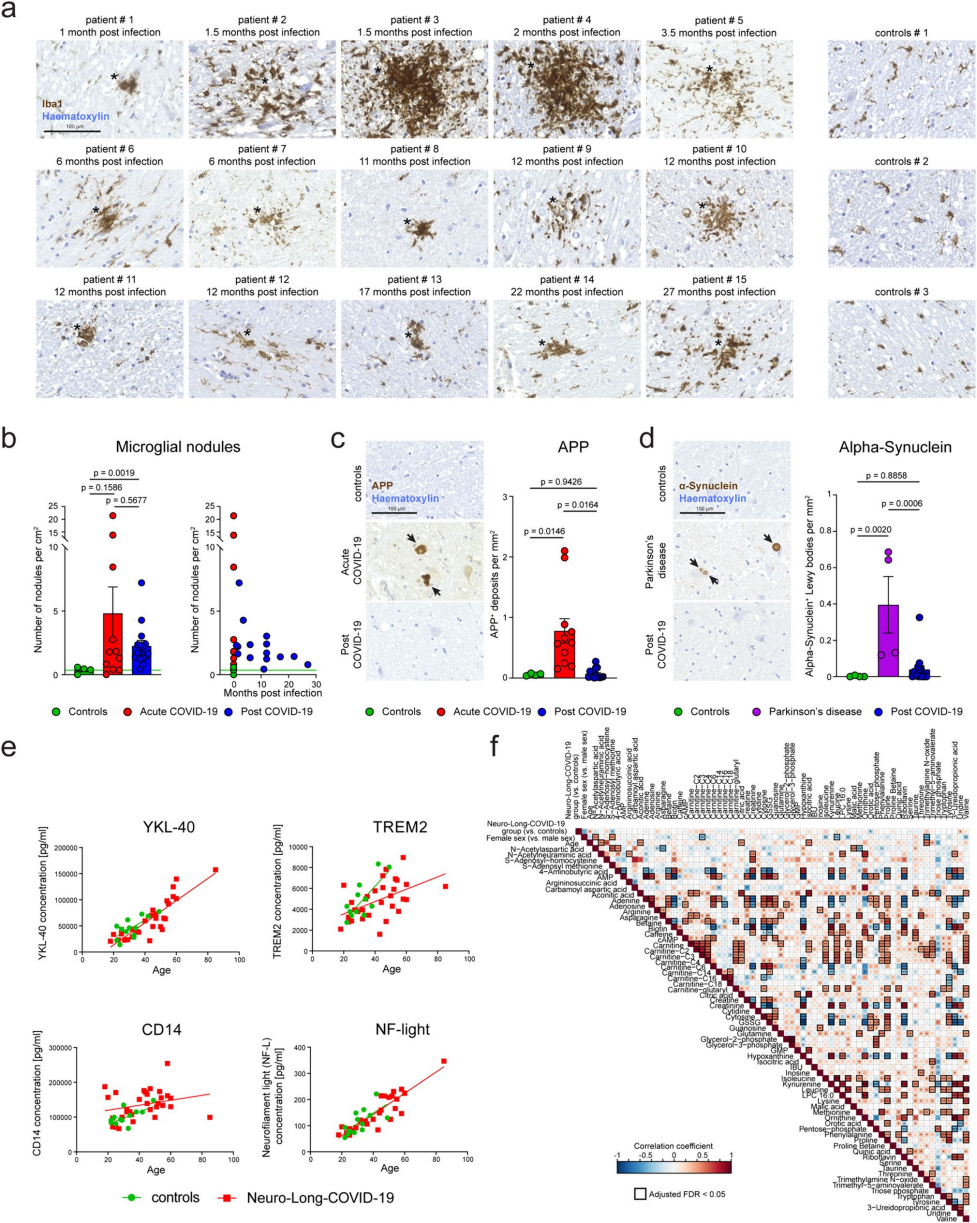
Persistent activation of microglia characterizes the CNS of post-COVID-19 patients. **a** Representative immunohistochemistry for Iba1 (brown) depicting typical microglial nodules (asterisks) in various medulla oblongata samples from post-COVID-19 and control brain samples. Counterstaining with haematoxylin (blue). Scale bars: 100 µm. **b** Left: quantification of microglia nodules in the medulla of controls, acute COVID-19 and post-COVID-19 patients. *P* values were determined using Brown–Forsythe and Welsh ANOVA test with Dunnett’s T3 multiple comparisons test. Bars represent means ± SEM. Each symbol represents one patient. Right: quantification thereof. Acute COVID-19 patients and controls are plotted at the 0 month time point. Green line indicates the mean of controls. **c** Left: illustrative picture of amyloid precursor protein (APP, brown) immunohistochemistry for axonal damage in the upper medulla. Counterstaining with haematoxylin. Scale bar: 100 µm. Arrows indicate APP^+^ deposits. Right: quantification of APP deposition. Each symbol represents one patient. Bars represent means ± SEM. Brown–Forsythe and Welsh ANOVA test with Dunnett’s T3 multiple comparisons test were performed. *P* values are shown. **d** Left: typical immunohistochemistry for alpha-synuclein (brown) in the brain stems of controls, Parkinson’s disease patients (used as control), and post-COVID-19 patients. Scale bar: 100 µm. Arrows indicate alpha-synuclein deposits. Right: quantification thereof. Each symbol represents one patient. Bars represent means ± SEM. Ordinary one-way ANOVA with Tukey’s multiple comparisons test was applied. *P* values are shown. **e** Protein levels of soluble YKL-40, TREM2, CD14, and neurofilament light chain (NF-light) measures by enzyme-linked immunosorbent assay (ELISA) in cerebrospinal fluid samples of Neuro-Long-COVID-19 patients and Post-COVID controls are shown. Each symbol represents one patient. Linear regression lines for each group are depicted. **f** Heatmap depicting CSF metabolites measured by targeted metabolomics. Colors indicate Spearman correlations of cerebrospinal fluid metabolites. Significance levels are indicated by asterisks (*P < 0.05, **P < 0.01, ***P < 0.001). Boxes show an adjusted FDR < 0.05

After having identified microglia nodules as the main immune feature of post-COVID-19 brains, we next asked for the chronicity and the functional relevance of this phenomenon, as these structures are usually absent in healthy brain tissue. Importantly, we found microglia nodules widespread present among post-COVID-19 brains compared to controls (Fig. 2a). Upon quantification, the number of microglia nodules was significantly higher in post-COVID-19 patients when compared to controls, but less frequent compared to acute COVID-19 brains (Fig. 2b). SARS spike glycoprotein immunohistochemistry did not reveal positive signal in the brain parenchyma, indicating the absence of viral presence (Supplementary Fig. 2). To evaluate the consequences of chronic microgliosis, we then assessed the extent of neuronal damage using immunohistochemistry for the amyloid precursor protein (APP), a surrogate marker for axonal damage (Fig. 2c). Although a significant increase in APP deposits was evident in acute COVID-19 cases, only individual patients in the post-COVID cohort exhibited deposits without reaching statistical significance for this group. Because COVID-19 may predispose individuals to develop Parkinson’s disease later in life [25], we investigated the cohort for the presence of alpha-synuclein deposits. In post-COVID-19 brains, we did not observe a significant increase of alpha-synuclein aggregates, a hallmark of several neuropathological conditions that show microglia nodules such as Parkinson’s disease (PD), dementia with Lewy Bodies (DLB), multiple system atrophy (MSA), and others [11] (Fig. 2d). In sum, obvious neuropathological correlates of neurodegeneration were absent from the investigated post-COVID-19 brains even at later stages.

To determine whether the histologically detectable persistent innate immune activation in post-COVID-19 brains is mirrored by any alterations in the cerebrospinal fluid (CSF), we analyzed this fluid compartment from 31 living individuals with clinically confirmed Neuro-Long-COVID-19 and respective controls (Supplementary Table 2). Neuro-Long-COVID-19 patients fulfilled the Post-COVID condition (PCC) criteria according to the WHO [23]. A few proteins have emerged as robust markers to monitor neuroinflammation in Alzheimer’s disease (AD) or multiple sclerosis due to their reproducible relation to pathological features of the disease: soluble TREM2 (sTREM2) as a marker of microglial activation [14], YKL-40 as an astroglia stimulation molecule [8], CD14 as myeloid cell activation protein [16] and neurofilament light chain (NF-light) as a correlate of neuronal damage. Notably, apart from the expected age-dependent increase, we did not observe higher levels of these markers in the clinically affected cohort compared to controls (Fig. 2e). Given the fact that microglia are metabolically active cells [7] that are extremely sensitive and versatile responders to minute changes of their microenvironment, we performed high-dimensional targeted metabolomics of the CSF and analyzed 67 metabolites in Neuro-Long-COVID-19 patients (Fig. 2f). Although typical metabolites associated with microglial activation, such as tryptophan, kynurenine, or glutamine, were clearly detectable, no major differences in the analyzed metabolite-levels between Neuro-Long-COVID-19 samples and controls were found using this highly sensitive method.

## Discussion

Taken together, by combining high-dimensional histological CyTOF analyses with machine learning methods, we studied the complexity of the brain immune landscape after systemic COVID-19 infections at the single-cell level. In this study, we examined autopsy cases from COVID-19 survivors at different time points after SARS-CoV-2 challenge. Long-term neurological symptoms had not been reported in this cohort. Since an autopsy cohort of Neuro-Long-COVID-19 patients was not available to us at this time, we have analyzed cerebrospinal fluid from living individuals with clinically confirmed Neuro-Long-COVID-19 as the closest approximation. Patients in the autopsy cohort had reported full recovery from COVID-19. They were typically tested negative for SARS-CoV-2 after their COVID-19 infection during their lifetime. Based on neuropathological analyses of the autopsy tissue, typical neuropathological hallmarks of neuronal degeneration were not detectable in this patient cohort. Nevertheless, we observed a clear shift from the T-cell linked adaptive immune activation during acute COVID-19 expositions toward a pronounced local innate immune stimulation in the CNS following virus resolution in this cohort. Our data further suggest a pervasive local pro-inflammatory milieu upon transient SARS-CoV-2 challenge mirrored by the presence and perseverance of microglia nodules.

In a parallel approach, we analyzed a cohort of living patients with clinically confirmed Neuro-Long-COVID-19 according to the WHO's Post-COVID Condition (PCC) criteria. Using cerebrospinal fluid (CSF) samples from these patients, we employed ELISA and targeted metabolomics to investigate potential disease-specific patterns. However, we could not detect a distinct disease-specific pattern in these analyses.

Taken together, we observed a dysregulation of the innate immune system in the autopsy cohort of COVID-19 survivors who did not present with neurological symptoms during their lifetime. This dysregulation might also be apparent in COVID-19 survivor with long-term neurological symptoms (Neuro-Long-COVID-19), potentially playing a role in the disease pathogenesis. However, establishing a definitive link remains challenging due to the absence of a dedicated autopsy cohort of patients with confirmed Neuro-Long-COVID-19 at this time. Further studies are required to explore this aspect in the future.

### Supplementary Information

Below is the link to the electronic supplementary material.

Supplementary file1 (PDF 5966 kb)
