# Supplementary material for: High throughput spatial immune mapping reveals an innate immune scar in post-COVID-19 brains
Source: Acta Neuropathol. 2024 Jul 25;148(1):11. doi: 10.1007/s00401-024-02770-6 (PMC11281987; doi:10.1007/s00401-024-02770-6)
Supplement: Supplementary file 1 — Supplementary file1 (PDF 5966 kb) [file 401_2024_2770_MOESM1_ESM.pdf]

## Supplementary Figure 1

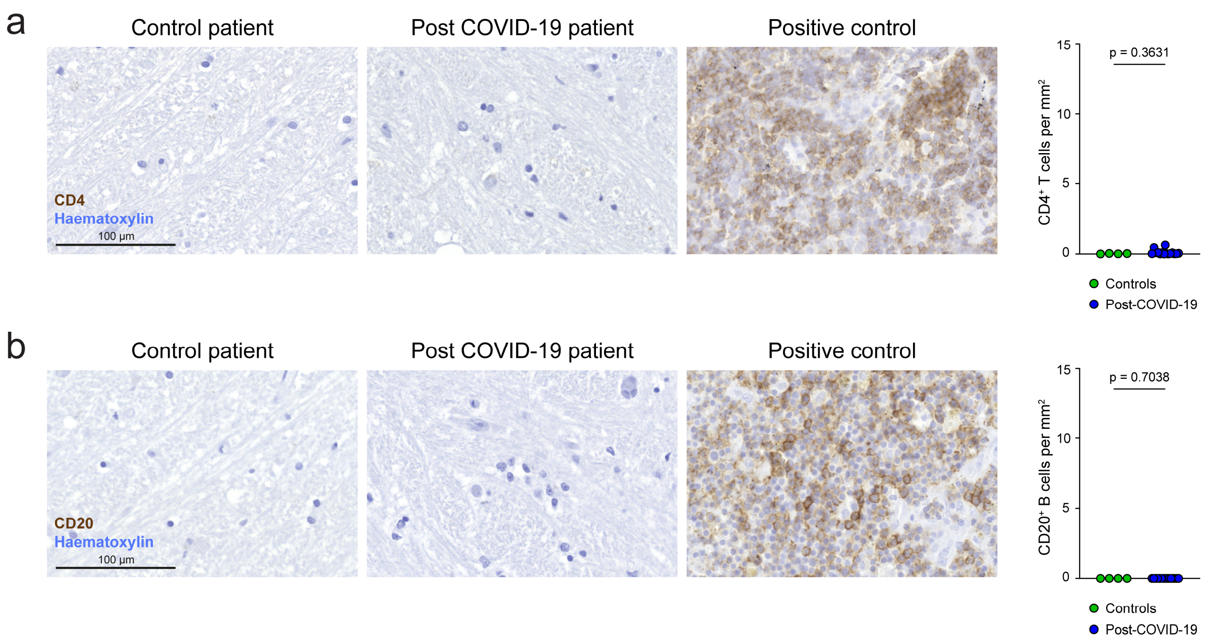


**Supplementary Figure 1: Examination of $CD4^+$ T cells and $CD20^+$ B cells in control and post COVID-19 brain specimens**

Immunohistochemical reactions for CD4 (a, brown) and CD20 (b, brown) in the upper medulla of a control and a post COVID-19 patient are depicted. Counterstaining with haematoxylin. Scale bar: 100 µm. The right panels show the parenchymal cell counts for each marker. Statistical analysis was performed using Student's t-test. *P* values are indicated in the figure. Each dot represents one patient.

## Supplementary Figure 2

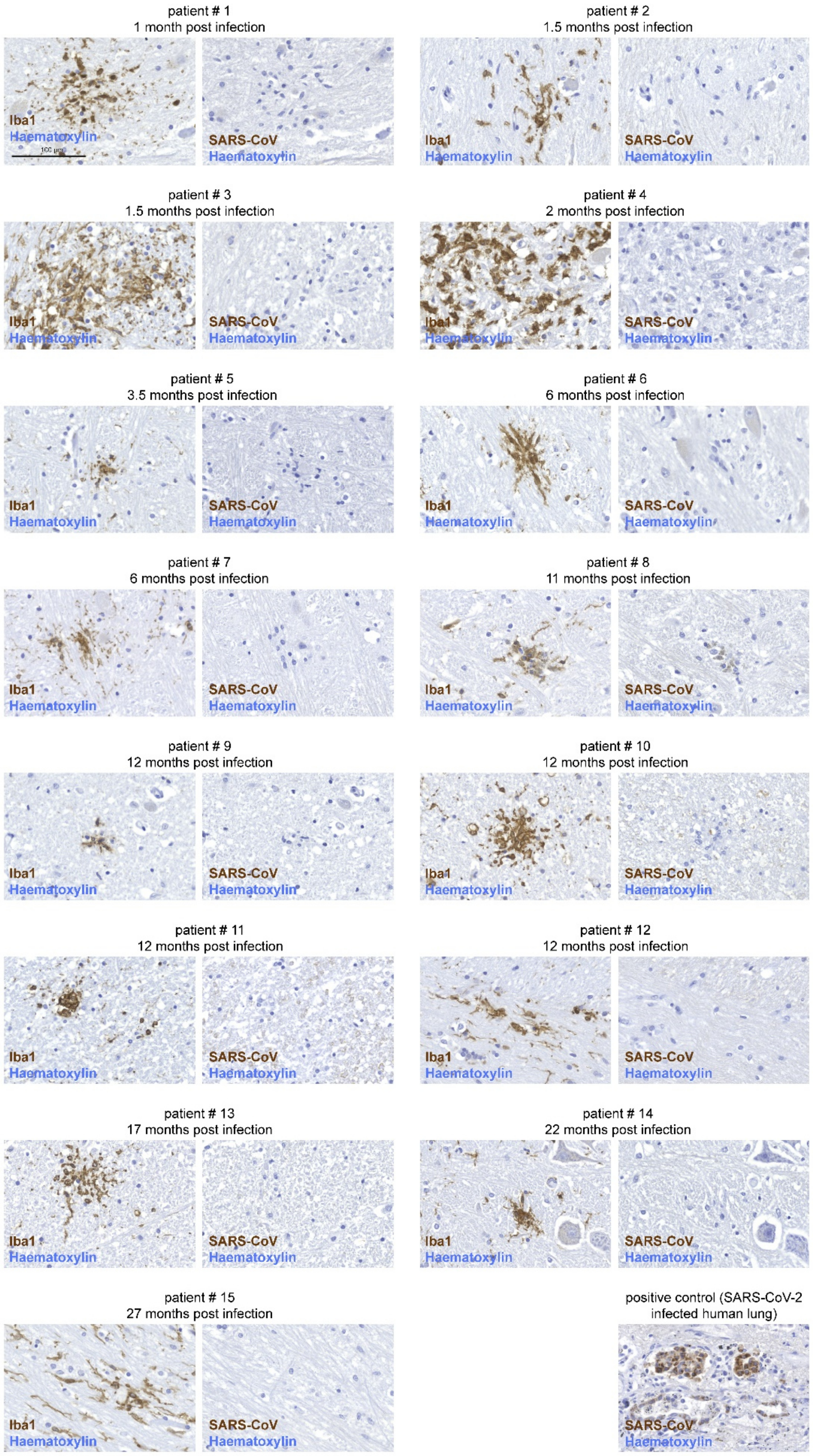

**Supplementary Figure 2: Immunohistochemical assessment of SARS spike glycoprotein in Post COVID-19 brainstem samples.**

For each patient, Iba1 immunohistochemistry of the medulla from post COVID-19 patients is depicted on the left, while consecutive sections stained with SARS spike glycoprotein are shown on the right. Scale bar: 100 µm. Counterstaining was performed with haematoxylin. Below on the far right, a positive control of SARS-CoV-2 infected human lung tissue is depicted.

## Supplementary Table 1

| Sample ID | Post COVID-19 survival interval | Sex | Age | Cause of death | Comorbidities | Brain weight (g) | macroscopic finding |
|---|---|---|---|---|---|---|---|
| Patient post COVID-19 # 1 | 1 month | male | 64 | Respiratory failure | status post myocardial infarct, Parkinson disease, LBD, lung emphysema | 1420 | none |
| Patient post COVID-19 # 2 | 1.5 months | female | 86 | Cardiac failure | metabolic syndrome, ceacal tubulovillous adenoma, adipositas | 1155 | cerebellar infarct foci bilateral (max. 1 cm); moderate brain edema |
| Patient post COVID-19 # 3 | 1.5 months | female | 79 | Respiratory failure | chronic renal insufficiency | 1120 | multiple foci of encephalomalacia (cerebellar, frontal right, striatal, frontal WM) |
| Patient post COVID-19 # 4 | 2 months | male | 49 | Cardiac failure | status after CPR, atherosclerosis, arterial hypertension, adipositas | 2470 | brain edema, focal pontine hemorrhage right |
| Patient post COVID-19 # 5 | 3.5 months | male | 63 | Cardiac failure | hypertension, no other comorbidities | 1500 | none |
| Patient post COVID-19 # 6 | 6 months | male | 83 | Cardiac failure | atherosclerosis, NPH, mixed dementia with Alzheimer and vascular changes, adipositas | 1120 | encephalomalacia frontal right (0.5 cm) |
| Patient post COVID-19 # 7 | 6 months | female | 51 | Cardiac failure | hypertension, asthma, coronary insufficiency | 1500 | none |
| Patient post COVID-19 # 8 | 11 months | female | 63 | Respiratory failure | Multiple Sclerosis (dx 1999, w/o therapy), AML | 1190 | none |
| Patient post COVID-19 # 9 | 12 months | male | 51 | Respiratory failure | lung adenocarcinoma | 1620 | none |
| Patient post COVID-19 # 10 | 12 months | male | 78 | Bolus death | chronic renal insufficiency, dementia | 1390 | atrophic brain, fibrotic arachnoidea |
| Patient post COVID-19 # 11 | 12 months | female | 79 | Septic shock | asthma, hypertension, diabetes mellitus, sarcoidosis, cardiac insufficiency, mammary carcinoma | 1240 | atrophic brain, film-like subarachnoidal and subdural hemorrhages, old cerebellar ischemic stroke |
| Patient post COVID-19 # 12 | 12 months | male | 89 | Cardiac failure | hypertension, chronic heart disease, dementia, prostate carcinoma, history of stroke, pulmonary emphysema | 1385 | old parietal ischemic stroke |
| Patient post COVID-19 # 13 | 17 months | male | 62 | Abdominal aortic dissection | no pre-existing conditions | 1560 | none |
| Patient post COVID-19 # 14 | 22 months | female | 55 | Cardiac failure | asthma, pericarditis | 1250 | none |
| Patient post COVID-19 # 15 | 27 months | female | 56 | Intestinal ischemia | IBD, adipositas with bariatric surgery > 10 yrs ago | 1295 | none |
| Patient with acute COVID-19 # 1 | - | male | 75 | Sudden cardiac death | Parkinson's disease | 1110 | none |
| Patient with acute COVID-19 # 2 | - | female | 85 | Pneumonia | atrial fibrillation, cardiac insufficiency, ischemic heart disease, myelofibrosis, renal insufficiency | 1240 | fresh infarction in territory of posterior cerebral artery |
| Patient with acute COVID-19 # 3 | - | female | 86 | Pneumonia | chronic obstructive pulmonary disease, dementia, ischemic heart disease | 1250 | fresh infarction in territory of posterior cerebral artery |
| Patient with acute COVID-19 # 4 | - | male | 90 | Pneumonia | atrial fibrillation, dementia, diabetes mellitus, history of stroke | 1015 | old infarctions in territory of posterior cerebral artery |
| Patient with acute COVID-19 # 5 | - | male | 70 | Pneumonia (aspiration) | cardiac insufficiency, chronic obstructive pulmonary disease, ischemic heart disease, Parkinsons's disease | 1430 | none |
| Patient with acute COVID-19 # 6 | - | male | 93 | Pneumonia | diabetes mellitus, hypertension | 1400 | none |
| Patient with acute COVID-19 # 7 | - | female | 54 | Pneumonia | trisomy 21, epilepsy | 950 | grey matter heterotopia |
| Patient with acute COVID-19 # 8 | - | female | 85 | Pneumonia | cardiac insufficiency, chronic obstructive pulmonary disease | 1180 | none |
| Patient with acute COVID-19 # 9 | - | female | 70 | Pneumonia | cardiac insufficiency | 1150 | none |
| Patient with acute COVID-19 # 10 | - | male | 93 | Pneumonia | atrial fibrillation, cardiac insufficiency, ischemic heart disease, obstructive sleep apnoea syndrome | 1000 | old cerebellar infarction |

| | | | | | | | |
|---|---|---|---|---|---|---|---|
| Patient with acute COVID-19 # 11 | - | female | 82 | Purulent bronchitis | chronic obstructive pulmonary disease, history of pulmonary embolism, renal insufficiency | 1080 | none |
| Controls # 1 | No history of SARS-CoV-2 infection | female | 75 | Multi-organ failure | B cell lymphoma, pneumonia, pericarditis, epicarditis | 1450 | none |
| Controls # 2 | No history of SARS-CoV-2 infection | male | 66 | Multi-organ failure | arterial hypertension, diabetes mellitus, atrial fibrillation, B cell lymphoma, T cell lymphoma, peripheral artery occusive disease, papillary renal cell carcinoma | 1600 | none |
| Controls # 3 | No history of SARS-CoV-2 infection | male | 64 | Cardiac failure | esophageal carcinoma, arterial hypertension, acute kidney failure, ARDS | 1500 | none |
| Controls # 4 | No history of SARS-CoV-2 infection | male | 78 | Hemorrhagic shock | arterial hypertension, depression, diabetes mellitus, coronary heart disease, benign prostate hyperplasia | 1250 | none |

**Supplementary Table 1: Characteristics of patients for post-mortem tissue analyses.**
The table displays the characteristics of the patients in the study.

## Supplementary Table 2

| Patient ID | Sex | Age | Group |
|---|---|---|---|
| # 1 | male | 43 | Neuro-Long-COVID-19 |
| # 2 | female | 58 | Neuro-Long-COVID-19 |
| # 3 | female | 25 | Neuro-Long-COVID-19 |
| # 4 | male | 47 | Neuro-Long-COVID-19 |
| # 5 | male | 58 | Neuro-Long-COVID-19 |
| # 6 | female | 44 | Neuro-Long-COVID-19 |
| # 7 | female | 36 | Neuro-Long-COVID-19 |
| # 8 | female | 34 | Neuro-Long-COVID-19 |
| # 9 | female | 22 | Controls |
| # 10 | female | 23 | Neuro-Long-COVID-19 |
| # 11 | female | 38 | Controls |
| # 12 | female | 34 | Controls |
| # 13 | female | 49 | Controls |
| # 14 | female | 25 | Controls |
| # 15 | female | 32 | Neuro-Long-COVID-19 |
| # 16 | male | 32 | Neuro-Long-COVID-19 |
| # 17 | female | 30 | Controls |
| # 18 | female | 85 | Neuro-Long-COVID-19 |
| # 19 | female | 29 | Controls |
| # 20 | female | 18 | Neuro-Long-COVID-19 |
| # 21 | male | 25 | Neuro-Long-COVID-19 |
| # 22 | female | 20 | Neuro-Long-COVID-19 |
| # 23 | male | 23 | Neuro-Long-COVID-19 |
| # 24 | male | 40 | Neuro-Long-COVID-19 |
| # 25 | male | 60 | Neuro-Long-COVID-19 |
| # 26 | male | 27 | Neuro-Long-COVID-19 |
| # 27 | male | 53 | Neuro-Long-COVID-19 |
| # 28 | male | 54 | Neuro-Long-COVID-19 |
| # 29 | male | 47 | Neuro-Long-COVID-19 |
| # 30 | female | 60 | Neuro-Long-COVID-19 |
| # 31 | female | 22 | Controls |
| # 32 | male | 52 | Neuro-Long-COVID-19 |
| # 33 | female | 35 | Neuro-Long-COVID-19 |
| # 34 | female | 28 | Controls |
| # 35 | female | 24 | Controls |
| # 36 | male | 29 | Neuro-Long-COVID-19 |
| # 37 | female | 50 | Neuro-Long-COVID-19 |
| # 38 | female | 51 | Neuro-Long-COVID-19 |
| # 39 | female | 33 | Controls |
| # 40 | female | 42 | Controls |
| # 41 | male | 45 | Neuro-Long-COVID-19 |
| # 42 | male | 45 | Neuro-Long-COVID-19 |
| # 43 | male | 55 | Neuro-Long-COVID-19 |
| # 44 | female | 33 | Controls |

**Supplementary Table 2: Characteristics of patients for cerebrospinal fluid analyses.**
The table displays the characteristics of the patients in the study.

## Supplementary Table 3

| Channel | Epitope | Manufacturer | Catalog | Clone | Dilution |
|---|---|---|---|---|---|
| 89 Y | CD45 | Atlas | AMAb90518 | CL0159 | 1:100 |
| 113 In | CD20 | BD | 555677 | H1 | 1:50 |
| 115 In | HLA-DR | abcam | ab176408 | TAL 1B5 | 1:400 |
| 141 Pr | MBP | abcam | ab230378 | EPR21188 | 1:100 |
| 142 Nd | Beta-Amyloid | Biolegend | 803001 | 6E10 | 1:100 |
| 143 Nd | GFAP | abcam | ab218309 | EPR1034Y | 1:200 |
| 144 Nd | INPP5D | Santa Cruz | sc-8425 | P1C1 | 1:100 |
| 145 Nd | Olig2 | R&D | AF2418 | polyclonal | 1:100 |
| 146 Nd | TYROBP | Atlas | HPA041899 | polyclonal | 1:400 |
| 147 Sm | CD163 | Novus | NB110-40686 | EDHU-1 | 1:400 |
| 148 Nd | NeuN | Biolegend | 834501 | 1B7 | 1:400 |
| 149 Sm | SMA | abcam | ab220795 | EPR5368 | 1:1600 |
| 150 Nd | TAU | Invitrogen | MN1020 | AT8 | 1:1600 |
| 150 Nd | PD-L1 | CST | 13684S | E1L3N | 1:100 |
| 151 Eu | CD31 | Fluidigm | 3151025D | EPR3094 | 1:200 |
| 152 Sm | Ki-67 | Invitrogen | 14-5698-82 | SolA15 | 1:800 |
| 153 Eu | FCERI | Millipore | 06-727 | polyclonal | 1:400 |
| 154 Sm | APPA4 | Merck | MAB348 | 22C11 | 1:400 |
| 155 Gd | P2RY12 | Atlas | HPA014518 | polyclonal | 1:800 |
| 156 Gd | CD4 | Abcam | ab181724 | EPR6855 | 1:200 |
| 157 Gd | CD64 | Sino Biological | 50086-R008 | 008 | 1:100 |
| 158 Gd | MX1 | abcam | ab284604 | EPR24485-19 | 1:400 |
| 159 Tb | CD68 | Biolegend | 916104 | KP1 | 1:1600 |
| 160 Gd | HLA-DRA | ProteinTech | 17221-1-AP | polyclonal | 1:200 |
| 161 Dy | Iba1 | abcam | ab220815 | EPR16588 | 1:1600 |
| 162 Dy | CD8a | Biolegend | 372902 | C8/144B | 1:800 |
| 163 Dy | TMEM119 | Sigma | AMab91528 | CL8714 | 1:800 |
| 164 Dy | HLA-DRB1 | ProteinTech | 15862-1-AP | polyclonal | 1:100 |
| 165 Ho | PD-1 | CST | 86163BF | D4W2J | 1:100 |
| 166 Er | CD204 | Invitrogen | 14-9054-82 | J5HTR3 | 1:800 |
| 167 Er | CD11c | Proteintech | 60258-1-Ig | 2F1C10 | 1:200 |
| 169 Tm | SLC2A5 | Atlas | HPA005449 | polyclonal | 1:800 |
| 170 Er | CD3 | CST | 85061S | D7A6E | 1:800 |
| 171 Yb | SCAMP2 | Atlas | HPA014699 | polyclonal | 1:800 |
| 172 Yb | Collagen IV | Millipore | AB769 | polyclonal | 1:1600 |
| 173 Yb | CD162 | Biolegend | 328802 | KPL-1 | 1:400 |
| 174 Yb | CD74 | Biolegend | 326802 | LN2 | 1:200 |
| 175 Lu | CD206 | Atlas | AMAb90746 | CL0387 | 1:400 |
| 176 Yb | S100A9 | Atlas | AMAb91690 | CL11191 | 1:1600 |
| 194 Pt | HH3 | CST | 4499BF | D1H2 | 1:100 |

**Supplementary Table 3: Imaging Mass Cytometry panel.**
The table shows the details of the panel used for Imaging Mass Cytometry (IMC).
